# Endocan: A Biomarker for Hepatosteatosis in Patients with Metabolic Syndrome

**DOI:** 10.1155/2020/3534042

**Published:** 2020-04-01

**Authors:** Hande Erman, Engin Beydogan, Seher Irem Cetin, Banu Boyuk

**Affiliations:** ^1^Department of Internal Medicine, SBU Fatih Sultan Mehmet Education and Research Hospital, Turkey; ^2^Department of Radiology, SBU Taksim Education and Research Hospital, Turkey; ^3^Department of Internal Medicine, SBU Taksim Education and Research Hospital, Turkey

## Abstract

**Background:**

Nonalcoholic fatty liver disease (NAFLD) is one of the most common chronic liver diseases, which has recently been mentioned as an independent cardiovascular risk factor.

**Objectives:**

Endocan is a novel molecule of endothelial dysfunction. We aimed to evaluate the associations of serum endocan levels with the hepatic steatosis index (HSI), fatty liver index (FLI), and degrees of hepatosteatosis in patients with metabolic syndrome with NAFLD. *Design and Setting*. This cross-sectional prospective study was performed in the outpatient clinic of an internal medicine department.

**Methods:**

The study included 40 patients with metabolic syndrome with NAFLD as noted using hepatic ultrasound and 20 healthy controls. Secondary causes of fatty liver were excluded. FLI and HSI calculations were recorded. Serum endocan level values were obtained after overnight fasting.

**Results:**

Higher values of HSI and FLI were found in the NAFLD groups than in the control groups (*p* < 0.001). Five (12.5%) of 20 patients with liver steatosis had grade 1 liver steatosis, 15 (37.5%) patients had grade 2 liver steatosis, and 20 (50%) patients had grade 3 liver steatosis. Serum endocan levels were lower in patients with NAFLD compared with the healthy controls (146.56 ± 133.29 pg/mL vs. 433.71 ± 298.01 pg/mL, *p* < 0.001). ROC curve analysis suggested that the optimum endocan value cutoff point for NAFLD was 122.583 pg/mL (sensitivity: 71.79%, specificity: 90%, PPV: 93.3%, and NPV: 62.1%).

**Conclusion:**

Serum endocan concentrations are low in patients with NAFLD, and the optimum cutoff point is 122.583 pg/mL. HSI and FLI were higher in patients with NAFLD; however, there was no correlation with serum endocan.

## 1. Introduction

Metabolic syndrome is an increasing public health problem for developed countries. The shared pathogenic mechanisms between metabolic syndrome and obesity-related disorders force us to be more sensitive in analyzing this population due to potential cardio metabolic consequences [1].

Nonalcoholic fatty liver disease (NAFLD) has a prevalence of 25% worldwide and is described as the leading cause of chronic liver disease [2]. In a study based on the data of NHANES III, liver disease was found to be the third leading cause of death among persons with NAFLD [3]. Its prevalence is increasing in conjunction with metabolic syndrome due to engaged risk factors, which include high body mass index and abdominal obesity, type 2 diabetes, dyslipidemia (high triglycerides and/or low high-density-lipoproteins), age, male sex, and alcohol consumption [4–6].

NAFLD is a silent condition that encompasses nonalcoholic fatty liver (NAFL) and nonalcoholic steatohepatitis (NASH) [7]. Although ultrasound and magnetic resonance are helpful for the diagnosis, liver biopsy is still the gold standard [8]. New insights and diagnostic improvements in NAFLD such as transient elastography and FibroScan are exciting alternatives [9]. However, we targeted focusing on noninvasive, cheap, and useful biomarkers in clinical practice. In this way, the role of circulating biomarkers related to endothelial dysfunction and the severity of underlying liver disease need to be investigated. Thus, we tested circulating levels of serum endocan to determine whether they may improve the timely prediction of fat content of liver and NAFLD.

Endocan, previously known as endothelial cell specific molecule-1, is a soluble form of dermatan sulfate, which is expressed by vascular endothelial cells of the lung, liver, and kidney. Tumor necrosis factor, interleukin-1, and lipopolysaccharides are suggested regulators of endocan expression [10, 11]. Systemic inflammation and subclinical atherosclerosis have a trigger role in atherosclerotic cardiovascular disease and are associated with NAFLD [12].

In the light of data in the literature, only a minority of patients with NAFLD proceed to the fibrotic and cirrhotic stages and develop end-stage liver disease. However, it is already known that nearly 40% of patients with NAFLD die of cardiovascular complications. For this reason, our study targeted predicting the stage and presence of NAFLD using serum endocan levels in order to prevent associated risks as soon as possible.

## 2. Methods

### 2.1. Study Population

In this cross-sectional study, 40 patients with metabolic syndrome with NAFLD were assessed. The criteria used for the diagnosis of metabolic syndrome were those recommended by the National Cholesterol Education Program. Expert Panel on Detection, Evaluation, and Treatment of High Blood Cholesterol in Adults-Adult Treatment Panel III (NCEP-ATP III), which is defined as the presence of at least three of these components: (1) increased waist circumference (>102 cm for men and >88 cm for women), (2) elevated triglycerides (≥150 mg/dL) or use of triglyceride-lowering drugs, (3) low levels of high-density lipoprotein (HDL) cholesterol (<40 mg/dL in men and <50 mg/dL in women), (4) hypertension (≥130/≥85 mmHg) or use of antihypertensive drugs, and (5) fasting glucose (≥110 mg/dL) or use of antidiabetic drugs [13].

Patients were included if they had a diagnosis of NAFLD as noted using hepatic ultrasound. Participants with any of the following possible secondary causes of fatty liver were excluded from the current analyses: (1) excessive alcohol intake (alcohol consumption was ≥20 g/day for men and ≥10 g/day for women over the past 12 months), (2) positive antihepatitis C virus (HCV) or hepatitis B surface antigen (HBsAg), (3) history drug use for treating fatty liver (i.e., amiodarone, corticosteroids, methotrexate, or tamoxifen), or (4) the absence of other causes of liver dysfunction, such as autoimmune liver disease, primary sclerosing cholangitis, Wilson's disease, and hereditary hemochromatosis. Other exclusion criteria were the presence of ischemic heart disease, congenital heart disease, valvular heart disease, and neoplastic, inflammatory, and infectious diseases.

This study was performed according to the guidelines of the Declaration of Helsinki, and it was approved by the ethics review committee of our hospital.

### 2.2. Physical and Laboratory Measurements

Anthropometric measurements, including weight, height, and systolic/diastolic blood pressure (BP), were measured following standardized protocols. Participants' seated BP was measured twice every 5 min on the right arm after 5 min of rest with a sphygmomanometer. The mean of the two readings was used in data analysis. The body mass index (BMI) was calculated according to the weight (kg) divided by the square of height (meters). Waist circumflex (WC) was measured midway between the uppermost border of the iliac crest and the lower border of the costal margin.

Overnight fasting (at least 8 h) blood samples were collected from the antecubital vein of each individual. Biochemical measurements, including assessment of fasting plasma glucose, total cholesterol (TCH), triglycerides (TG), low-density lipoprotein cholesterol (LDL-C), HDL-C, alanine aminotransferase (ALT), aspartate aminotransferase (AST), gamma-glutamyltransferase (GGT), and glycated hemoglobin (HbA1C), were measured enzymatically on an auto analyzer (COBAS 311, Roche Diagnostics GmbH, Mannheim, Germany). Particle-enhanced immunoturbidimetry was performed using a Behring Nephelometer BN-100 (Behring Diagnostic, Frankfurt, Germany) which was used to measure C-reactive protein (CRP). The sensitivity of the test was 0.1 mg/L. Serum endocan levels were measured using a Sunred enzyme-linked immunosorbent assay (ELISA) kit (Sunred Biological Technologies Human ECSM1/ENDOCAN ELISA Kit, catalog No.: 201-12-1978, China). The intra- and interassay variabilities of the ELISA kit were 5.1% and 6.1%, respectively. The minimum detectable level of endocan was 31.2 pg/mL.

### 2.3. FLI and HSI Were Calculated on the Basis Sample Analyses

Hepatic steatosis index (HSI) = 8 ∗ ALT/AST + BMI (+2, if DM; +2, if female). At a value of <30.0, HSI could rule out steatosis. At a value of ≥36, HSI could rule in steatosis [14].

Fatty liver index (FLI) = logistic (0.953 ∗ ln (TG) + 0.139 ∗ BMI + 0.718 + ln (GGT) + 0.053 ∗ waist 15.745) ∗ 100, where logistic (x) = 1/(1 + ex) denotes the logistic function and ln the natural logarithm. Values< 30 rule out steatosis, and values ≥ 60 rule in steatosis [15].

### 2.4. Liver Ultrasound Measurements

Ultrasound evaluations were performed by a single radiologist using a 1-6 MHz PVT-375 BT convex transducer (Toshiba A500 Platinum). Ultrasound examination of the liver was performed after 12 hours fasting. Each subject was examined in the supine and left lateral positions during quiet inspiration and asked to stop breathing during inspiration. The presence or absence and grading of fatty infiltration of the liver were recorded. Hepatic steatosis was defined as the presence of an ultrasonography pattern of parenchymal brightness (from normal to severe increased), liver-kidney contrast (absent = 0/present = 1), deep beam attenuation (diaphragm bright and clear = 0/diaphragm blurred = 1), and bright vessel walls in the parenchyma (present = 0/absent = 1) [16].

### 2.5. Statistical Analysis

Frequency, ratio, mean, minimum, maximum, and standard deviation values were used in the descriptive statistics to determine continuous variables. Student's *t*-test was used for comparisons of two independent and normally distributed variables. The Mann-Whitney *U* test was performed for the comparison of independent and nonnormally distributed variables. The chi-squared test and Fisher's exact test were performed to determine differences between categorical variables. Multiple linear logistic regression analysis was performed to determine the effect levels of the parameters. Spearman's correlation tests were used for correlation analyses. Receiving operating characteristics (ROC) curve analysis was performed to define the sensitivity and specificity of serum endocan to predict NAFLD. Statistical significance was assessed at *p* < 0.05. Statistical analysis was performed using the MedCalc Statistical Software version 12.7.7 (MedCalc Software bvba, Ostend, Belgium; http://www.medcalc.org; 2013).

## 3. Results

The clinical characteristics of the study subjects are shown in Table 1. Sex and age distribution were similar between the groups. There were no significant differences in DBP, TCH, LDL-C, and AST levels between the patients with metabolic syndrome with NAFLD and control subjects. The patients with NAFLD had a significantly higher SBP, BMI, waist circumference, fasting plasma glucose, HbA1C, TG, ALT, GGT, and CRP levels than the control subjects (*p* < 0.01). It was observed that HDL-C levels were significantly lower in patients with NAFLD than in the controls (*p* < 0.05). The serum endocan levels were significantly lower in patients with NAFLD compared with the healthy controls (146.56 ± 133.29 pg/mL vs. 433.71 ± 298.01 pg/mL, *p* < 0.001). The HSI and FLI values were statistically significantly higher in the NAFLD groups than in the control group (*p* < 0.001).

Five (12.5%) of 20 patients with liver steatosis had grade 1 liver steatosis, 15 (37.5%) patients had grade 2 liver steatosis, and 20 (50.0%) patients had grade 3 liver steatosis.

The serum endocan levels showed a positive correlation with age (*r* = 0.309; *p* = 0.05) and a negative correlation with the BMI (*r* = −0.386; *p* < 0.015) in NAFLD, as shown in Table 2. However, there was no significant correlation between the other variables and endocan levels. Regression analysis of BMI and serum endocan revealed that exponentiated coefficient was found to be -40.43 which means one unit increase of BMI results in 40.43% decrease of serum endocan (not shown in table).

**Figure 1:**
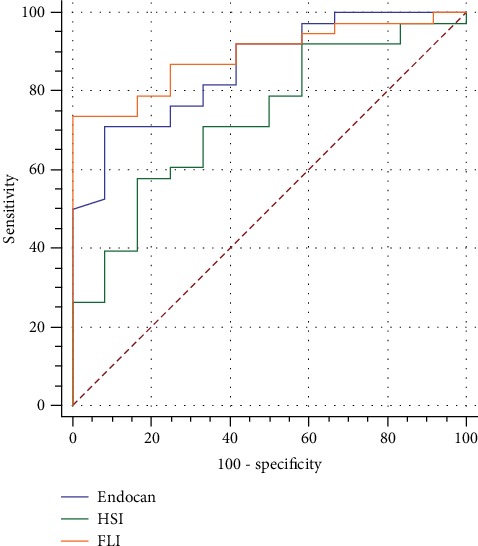
Sensitivity and specificity parameters for serum endocan, HSI, and FLI in patients of NAFLD. HSI: hepatic steatosis index; FLI: fatty liver index; NAFLD: nonalcoholic fatty liver disease.

ROC analysis and diagnostic screening tests were used to determine the cutoff point for endocan. Patients who had an endocan level lower than 122.583 pg/mL were indicated NAFLD with a sensitivity of 71.79% and a specificity of 90%. The cutoff value of serum endocan level to predict NAFLD was 122.583 pg/mL with a positive predictive value of 93.3% and a negative predictive value of 62.1%. The cutoff value of HSI was 16.76 to predict NAFLD (AUC = 0.893, *p* < 0.001). The cutoff value of FLI was 40.86 (AUC = 0.893, *p* < 0.001) (Table 3 and Figure 1). Furthermore, AUC values for endocan and HSI were similar (*p* = 0.219). Also, AUC values for endocan and FLI were statistically similar (*p* = 0.594). In other words, prediction power of endocan for fatty liver is similar with FLI and HSI. However, FLI predictive power for fatty liver is more than HSI (*p* = 0.048) (Table 4).

## 4. Discussion

In this present study, we demonstrated low serum concentrations of endocan in patients with metabolic syndrome with NAFLD in comparison with healthy individuals, which gives an insight into the role of endocan in NAFLD.

Patients with metabolic syndrome are prone to cardiovascular complications more frequently than the healthy population due to visceral obesity, defects in glucose metabolism, and endothelial dysfunction [17]. Recently, NAFLD was regarded as the hepatic manifestation of metabolic syndrome; however, it is still speculative as to which produces the latter [18]. Consequently, both NAFLD and metabolic syndrome have emerged as a growing health problem in developed countries [19]. Although the main trigger factor is insulin resistance in relation with metabolic syndrome, a genetic predisposition and unhealthy lifestyle can precipitate the development of fatty liver [20]. Other than insulin resistance, one of the key mechanisms recently mentioned is the endothelial dysfunction in the progression of NAFLD. It would seem that progression of NAFLD related to increase of active forms of oxygen and nitrogen which leads to a significant arterial vasospasm and a progressive damage to the endothelium [21]. Therefore, the cardiovascular consequences and related metabolic disorders require a multidisciplinary approach to the management of NAFLD.

NAFLD has a silent progress, and the majority of cases have an asymptomatic increase in AST and ALT. In general, diagnosis is suspected with abnormal liver function tests. Unfortunately, aminotransferases do not identify progressive disease. Ultrasonography, computed tomography, and magnetic resonance imaging of the liver are the standard imaging modalities used in clinical practice for diagnosis and detection disease progression [22]. Traditionally, liver biopsy is the gold standard for the diagnosis and staging of NAFLD; however, it is impractical to perform and hard to convince patients for the procedure [23]. Recent studies suggested that with the use of standardized measurements, it was possible to detect amounts of steatosis as little as 10% [24–26]. Besides radiologic evaluations, some indexes were developed using anthropometric and laboratory parameters in order to predict hepatosteatosis severity [27]. In our study, we found higher scores of FLI and HSI in patients with NAFLD than in healthy individuals.

Endocan, which is one of the endothelium-derived proteoglycans, has been suggested to be an indicator of endothelial activation in recent studies [28]. It has been shown to increase in patients with infectious disease, malignancy, cardiovascular disease, and type 2 diabetes [29–32]. In a study by Dallio et al., NAFLD was found to be associated with a significant increase in serum endocan levels when compared with healthy controls. However, it was later realized that this increase was more marked in steatohepatitis than in simple steatosis, independent from the presence of diabetes [33]. In our study, the mean level of serum endocan in healthy individuals was 433.71 pg/mL, similar to previous studies [34–36]. We found low serum endocan levels in patients with metabolic syndrome with fatty liver disease, which seems to conflict with some other studies [36]. However, due to lack of liver biopsy of the patient group, we could not demonstrate the ratio of steatohepatitis, which is more likely to be associated with endothelial dysfunction among patients with NAFLD.

In a similar manner, Ustyol et al. found that NAFLD had no additional influence on circulating serum endocan concentrations in adolescents with obesity, which is in accord with the findings of Janke et al. [28]. Janke et al. hypothesized that due to loss of a vasoprotective factor, obesity could be associated with decreased ESM-1 formation in adipose tissue and decreased endocan levels in plasma [37]. These data obtained from previous studies can clarify the negative correlation of the serum endocan levels and BMI of the patients in our study. The other parameter that showed a correlation with endocan was age. According to our results and those in previous studies, this positive correlation may be related with factors other than obesity.

To our knowledge, the relation between serum endocan and NAFLD has not yet been investigated in patients with metabolic syndrome. We demonstrated the predictive capacity of serum endocan regarding NAFLD in metabolic syndrome, with an AUROC of 0.867. For a patient with metabolic syndrome, serum endocan levels less than 122.583 pg/mL predict NAFLD with a sensitivity of 71.79% and a specificity of 90%. It had a positive predictive value of 93.3% and a negative predictive value of 93.3% (Table 3). Along with these data, for a patient with metabolic syndrome, the cutoff value of FLI to predict NAFLD was 40.86, and the cutoff value of HSI was 16.76 to predict NAFLD which were similar to the literature data [14, 15]. The comparison of serum endocan, HSI, and FLI in relation to predictive values, serum endocan was seem to have similar clinical value (Table 4). However, serum endocan gives an insight about endothelial dysfunction and inflammation in patients with NAFLD in metabolic syndrome which should be further addressed in clinical studies.

More importantly, the standardization and cutoff values of serum endocan have been controversial in the literature. For the first time, the current study suggests a cutoff point for serum endocan of NAFLD in patients with metabolic syndrome. However, there are some limitations of our study. The first limitation is the small sample size, which can serve as a pilot study. The second limitation is the lack of histologic confirmation of NAFLD; however, it is costly and impractical to perform in a disease with such a high prevalence. On the other hand, hepatic imaging is an acceptable assessment tool in NAFLD [7]. Finally, it has been suggested that the usefulness of FLI in mild hepatosteatosis is questionable, which may have led to underdiagnosis of fatty liver [15]. In our study, we used biochemical parameters, FLI, HSI, and ultrasound examinations in patients to increase the accuracy of the assessment of NAFLD severity.

In conclusion, NAFLD was predicted using SBP, BMI, waist circumference, fasting plasma glucose, HbA1C, TG, ALT, GGT, CRP, and serum endocan, along with the ultrasound images. Although serum endocan was found to lack a correlation with FLI and HSI, ROC analysis revealed a prediction of NAFLD at 122.583 pg/mL for the first time in metabolic syndrome. Nevertheless, it should be acknowledged that other novel markers beyond serum endocan will further clarify the prediction of NAFLD and the role of endothelial dysfunction-mediated metabolic complications in patients with NAFLD.

## Figures and Tables

**Table 1:** Clinical and laboratory characteristics of controls and patients of NAFLD.

	Patients of NAFLD (*n* = 40)	Control group (*n* = 20)	
	Mean ± s.d./*n*, %	Mean ± s.d./*n*, %	
Age (years)	54.05 ± 9.09	52.5 ± 7.98	
Gender			
Male	16 (40%)	10 (50%)	
Female	24 (60%)	10 (50%)	
BMI (kg/m^2^)	31.62 ± 4.65	25.58 ± 3.67	^∗∗^
Waist circumference(cm)	109.72 ± 11.73	85.05 ± 11.79	^∗∗^
Systolic pressure (mmHg)	132.05 ± 21.08	117.5 ± 9.25	^∗∗^
Diastolic pressure (mmHg)	73.97 ± 11.99	71.25 ± 8.72	
Fasting blood glucose (mg/dL)	171.59 ± 96.09	91.2 ± 12.48	^∗∗^
HbA1c (%)	8.42 ± 2.76	5.55 ± 0.46	^∗∗^
Triglyceride (mg/dL)	250.05 ± 198.03	115.1 ± 57.96	^∗∗^
Total cholesterol (mg/dL)	211.1 ± 49.91	207.1 ± 35.89	
LDL (mg/dL)	119.56 ± 36.27	129.1 ± 30.31	
HDL (mg/dL)	46.1 ± 10.81	54.95 ± 14.92	^∗^
CRP (mg/L)	6.1 ± 3.87	2.3 ± 2.02	^∗∗^
AST (U/L)	27.38 ± 17.34	19.75 ± 3.67	
ALT (U/L)	32.49 ± 25.53	19.05 ± 5.92	^∗^
GGT (U/L)	40.33 ± 31.72	20.6 ± 8.59	^∗∗^
Endocan (pg/mL)	146.56 ± 133.29	433.71 ± 298.01	^∗∗^
FLI	51.52 ± 24.7	6.57 ± 35.23	^∗∗^
HSI	33.9 ± 29.6	17.6 ± 7.1	^∗∗^
NAFLD grade			
0	0 (0%)	20 (100%)	
1	5 (12.5%)	0 (0%)	
2	15 (37.5%)	0 (0%)	
3	20 (50.0%)	0 (0%)	

Mann-Whitney *U*, Student's *t*, and Fisher's exact test. Statistical significance: ^∗^*p* < 0.05, ^∗∗^*p* < 0.01. BMI: body mass index; HOMA-IR: homeostasis model assessment of insulin resistance; LDL: low-density lipoprotein; HDL: high-density lipoprotein; CRP: C-reactive protein; AST: aspartate transaminase; ALT: alanine transaminase; GGT: gamma-glutamyl transpeptidase; FLI: fatty liver index; HSI: hepatic steatosis index.

**Table 2:** Correlation between endocan and characteristics in patients of NAFLD.

	Endocan
Age (years)	
*r*	0.309
*p*	0.05
BMI (kg/m^2^)	
*r*	-0.386
*p*	0.015
HSI	
*r*	-0.223
*p*	0.173
FLI	
*r*	-0.262
*p*	0.112

NAFLD: nonalcoholic fatty liver disease; BMI: body mass index; HSI: hepatic steatosis index; FLI: fatty liver index.

**Table 3:** ROC analysis and diagnostic screening tests were used to determine the cutoff point for endocan, HSI, and FLI.

Steatosis present vs. not present	AUC	*p* value	Cutoff	Sensitivity	Specificity	PPV+	PPV-
Endocan	0.867	<0.001	122.583	71.79	90.0	93.3	62.1
HSI	0.814	<0.001	>16.67	92.3	60.00	81.8	80.00
FLI	0.893	<0.001	>40.86	73.7	100.00	100.00	54.5

ROC: receiver operating system; HSI: hepatic steatosis index; FLI: fatty liver index; AUC: area under curve. Statistical significance: *p* < 0.05 and *p* < 0.01.

**Table 4:** The comparative assessment of AUC for endocan, HSI, and FLI.

	HSI (AUC difference) *p* value	FLI (AUC difference) *p* value
Endocan	(0.130)	(0.031)
	0.219	0.594
HSI		(0.162)
		0.048

HSI: hepatic steatosis index; FLI: fatty liver index; AUC: area under curve.

## Data Availability

The data used to support the findings of this study are available from the corresponding author upon request.
